# One-year survival of minimally invasive restorations in patients with disabilities: implications for clinical decision-making in special care dentistry

**DOI:** 10.3389/froh.2026.1846405

**Published:** 2026-05-20

**Authors:** Mariana Ruth Zar, Leandro Navarro, Doriana Torres, Gustavo Fabián Molina

**Affiliations:** Special Care Dentistry, The Dental School, Faculty of Health Sciences, Universidad Católica de Córdoba, Córdoba, Argentina

**Keywords:** atraumatic restorative treatment, disability, minimally invasive dentistry, restorative survival, silver diamine fluoride, special care dentistry

## Abstract

**Background:**

Unmet caries treatment needs are common among people with disabilities, in part due to difficulties with cooperation during conventional dental care. This cohort study aimed to compare the acceptability, feasibility, and survival of Atraumatic Restorative Treatment (ART), Silver-modified Atraumatic Restorative Treatment (SMART), and Conventional Restorative Treatment (CRT) in patients with disabilities referred to a university dental clinic.

**Methods:**

Seventy-four patients (mean age 20.6 ± 11.2 years) referred for restorative care chose: ART (*n* = 26), SMART (*n* = 35), CRT (*n* = 11); two others required sedation/general anesthesia. A total of 100 restorations were placed by one operator and assessed by two calibrated independent evaluators (Kappa = 0.80) at 6 and 12 months. The restorative material selected for ART and SMART was Fuji 9 Hybrid (GC America, Alsip US) whereas G-Bond + Gaenial Accord were used for CRT (GC America, Alsip US). Acceptability, feasibility, and complexity of care were determined using BDA scores and the iADH uCMT domains. Statistical analyses included Chi-square tests, Kaplan–Meier survival analysis, and Cox regression (α = 0.05). Spearman correlation was used to assess the association between care complexity and BDA and iADH uCMT scores.

**Results:**

ART, SMART, and CRT were feasible for all patients selecting those options, with optimal restoration placement in 100% of ART and CRT cases, and 95.8% in SMART. Complexity scores (BDA) were significantly higher in the SMART group (OR = 3.730; 95% CI: 1.82–6.75; *p* ≤ 0.001). The “Communication” and “Behavioral Support” domains required fewer adaptations for CRT patients (*p* = 0.001), whereas “Access to adapted care” required more adaptations in ART and SMART groups. Out of 100 restorations, 26 failed by ART criteria and 23 by USPHS criteria. According to the latter, survival rates were 90.3% for ART, 67.4% for SMART, and 84.3% for CRT, with significant differences between ART/CRT vs. SMART (*p* < 0.001).

**Conclusions:**

ART and SMART are acceptable and feasible restorative approaches for patients with disabilities, particularly when adaptations across care domains are required. Patients with lower BDA scores are more likely to opt for CRT. However, the significantly lower survival associated with SMART challenges its suitability as a durable option in this population.

## 1 Introduction

Oral health is a fundamental component of general health and quality of life (1). Despite global advances, oral diseases remain among the most prevalent non-communicable conditions, particularly affecting vulnerable populations such as individuals with disabilities (2). Functional limitations, behavioral challenges, and restricted access to care contribute to significant unmet treatment needs in this group (1, 3).

Minimally invasive dentistry has been proposed as a paradigm shift toward more patient-centered and accessible care (4). Techniques such as Atraumatic Restorative Treatment (ART) (5) and Silver-Modified Atraumatic Restorative Treatment (SMART) (6) aim to preserve tooth structure while reducing procedural complexity, discomfort, and aerosol generation—factors particularly relevant in Special Care Dentistry and during public health crises such as the COVID-19 pandemic (7).

ART relies on selective caries removal using hand instruments and restoration with high-viscosity glass ionomer cement (GIC), a material known for its chemical adhesion and fluoride release (8). SMART combines the cariostatic effect of silver diamine fluoride (SDF) with GIC restoration, potentially enhancing caries control while maintaining minimal invasiveness (9).

Although both approaches have demonstrated promising results, evidence regarding their long-term effectiveness—especially in patients with disabilities—remains limited and sometimes conflicting. Furthermore, treatment selection in this population is strongly influenced by patient complexity, caregiver preferences, and accessibility constraints (10).

Within the context of emerging frameworks aimed at transforming Special Care Dentistry through clinical innovation and personalized care pathways, evaluating the real-world performance of these techniques is essential.

Therefore, the aim of this cohort study was to compare the acceptability, feasibility, and survival of ART, SMART, and Conventional Restorative Treatment (CRT) in patients with disabilities treated in a university-based dental clinic, and to explore their implications for clinical decision-making in Special Care Dentistry.

## 2 Materials and methods

A preliminary article regarding acceptability and feasibility of minimally invasive treatment for the present cohort study was published and materials and methods were thoroughly described elsewhere (10). Nevertheless, and for the purpose of clarity for the reader, here is a shortened version for reference.

### 2.1 Study design and ethical approval

This observational cohort study was conducted in accordance with the Declaration of Helsinki and approved by the Institutional Ethics Committee of the School of Dentistry, Catholic University of Córdoba (protocol ODON20230906bP). The study design followed the framework of a previously registered clinical trial (Netherlands Trial Register No. 4400).

### 2.2 Participants

Participants with disabilities, as defined by the International Classification of Functioning, Disability and Health (ICF), were recruited between August 2023 and July 2024 at a university-based dental clinic for Special Care Dentistry. In ICF terms, a “patient with a disability” would be understood as “an individual who experiences impairments in body functions or structures, limitations in activities, or restrictions in participation, arising from the interaction between their health condition and contextual (environmental and personal) factors”.

Individuals of any age or sex presenting with at least one carious lesion classified as ICDAS 3–5 in primary and/or permanent teeth, without signs of pulpal involvement or tooth mobility, and in functional occlusion were considered eligible.

Written informed consent was obtained from all participants or their legal guardians. One of two trained dentists in Special Care Dentistry clinically examined participants who met the inclusion criteria and consented to participate.

### 2.3 Clinical assessment

Baseline clinical evaluation included (Table 1):

1. Assessment of pain reported by the participant and/or caregiver and identification of symptomatic teeth;
2. Oral hygiene status, assessed using the Simplified Oral Hygiene Index (OHI-S) (11);
3. Gingival bleeding, recorded on buccal and lingual surfaces according to Ainamo and Bay criteria (12);
4. Caries experience, assessed using the DMFT/dmft index according to World Health Organization criteria (13), and caries detection using ICDAS II (14).

**Table 1:** Baseline oral risk factors in the population included for treatment.

Variable	Value
Total participants	74
Age (mean ± SD)	20.6 ± 11.2
Sex (F/M)	37/37
DMFT (mean ± SD)	15.8 ± 12.3
dmft (mean ± SD)	4.7 ± 7.5
OHI-S (mean ± SD)	2.5 ± 1.5
Plaque prevalence	100%
Gingival bleeding	100%

Mean caries experience was high (DMFT: 15.8 ± 12.3; dmft: 4.7 ± 7.5). All participants presented dental plaque and gingival bleeding (100%), with a mean simplified oral hygiene index (OHI-S) of 2.5 ± 1.5.

### 2.4 Assessment of care complexity

Although medical condition of all participants was recorded, functional status and disability using the ICF Checklist for Oral Health (15) was better reflected in the domains of the British Dental Association (BDA) Case Mix Tool (16) and complemented with the International Association for Disability and Oral Health (iADH) universal Case Mix Tool (uCMT) (17).

The complexity of care was assessed using the BDA Case Mix Tool (16), which evaluates six domains: communication ability, cooperation, medical status, oral risk factors, access to oral care, and legal/ethical barriers. Scores were categorized into five levels: standard (0), mild (1–9), moderate (10–19), severe (20–29), and extreme (≥30).

Additionally, the International Association for Disability and Oral Health (iADH) universal Case Mix Tool (uCMT) (17) was used to identify domains most strongly associated with treatment selection and required care adaptations.

Examiners were calibrated every 15 participants, achieving a Cohen's kappa coefficient of 0.85.

### 2.5 Information and treatment allocation

At the initial visit, the objectives and study design were thoroughly explained to all eligible participants and/or their caregivers (hereafter referred to as “respondents”). Information was provided in an accessible format to support shared decision-making, particularly for participants with intellectual disabilities.

The presence of carious lesions and the need for treatment were explained, and respondents were informed about the available treatment options both verbally and printed informational leaflets. These materials were content-validated by the Argentine Association of Disability and Oral Health and pilot-tested independently in Special Care Dentistry settings with individuals not involved in the study.

Respondents were allowed to review the information at home. At a second visit, those who agreed to participate and signed informed consent selected one of the following treatment approaches according to their preferences:

- Conventional Restorative Treatment (CRT)
- Atraumatic Restorative Treatment (ART)
- Silver-Modified Atraumatic Restorative Treatment (SMART)

**Figure 1:**
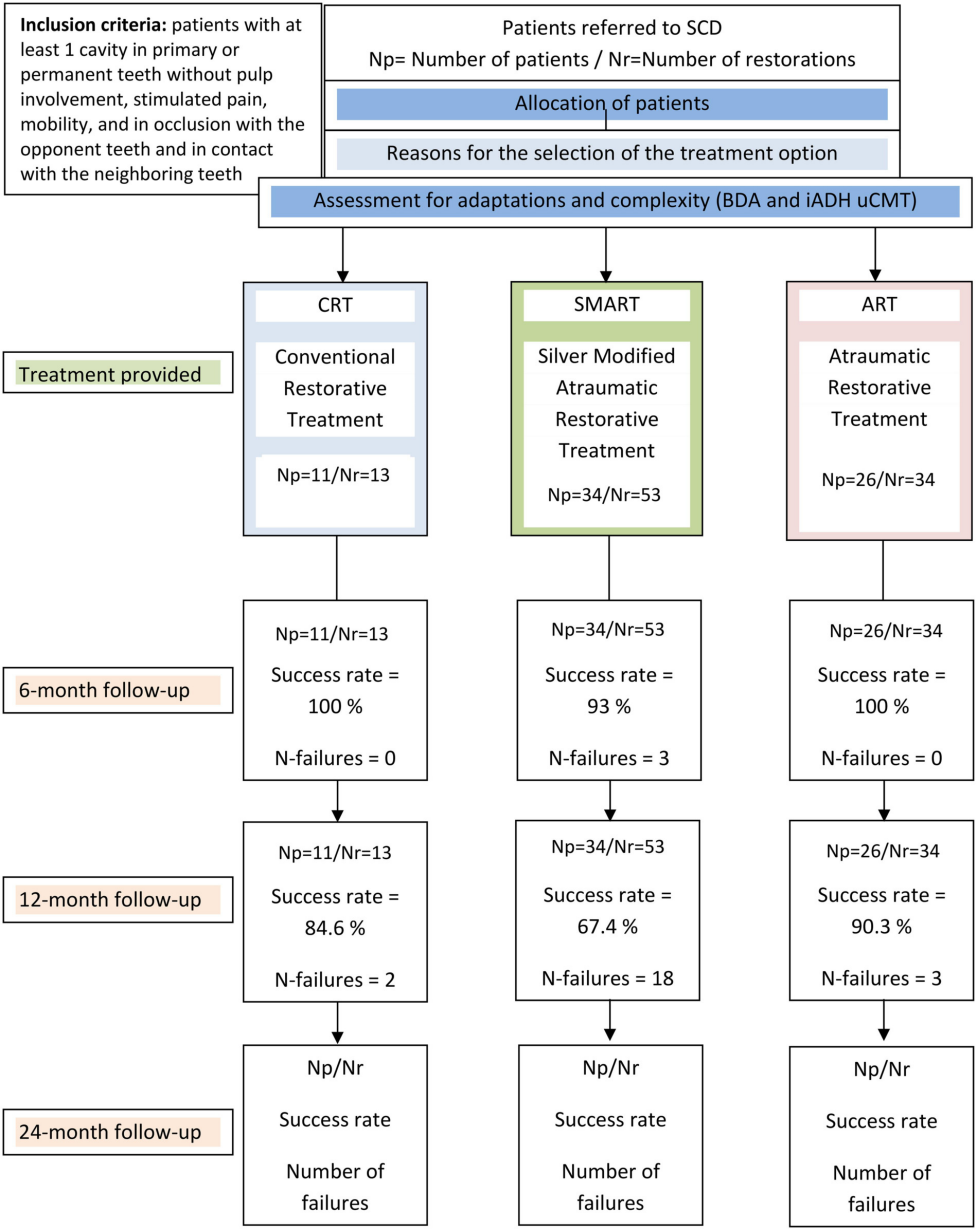
Flow-chart of the study.

Participants were then allocated to the corresponding treatment cohort (Figure 1).

### 2.6 Clinical procedures

A single trained operator, distinct from the examiners, following standardized protocols for each treatment approach, performed all procedures.

#### 2.6.1 Conventional restorative treatment (CRT)

Dentin carious lesions were treated under local anesthesia using high-speed rotary instruments and rubber dam isolation. Residual carious tissue was removed using low-speed burs. Deep cavities received calcium hydroxide liner when indicated.

Cavities were restored using an adhesive system (G-Bond, GC Tokyo, Japan) and nanohybrid composite resin (Gaenial ÁChord, GC Tokyo, Japan) applied incrementally and light-cured according to manufacturer instructions. Finishing and polishing were performed using standard rotary instruments and polishing systems.

#### 2.6.2 Atraumatic restorative treatment (ART)

Selective removal of soft, demineralized carious dentin was performed using hand instruments only, following the ART protocol (18).

Cavities were conditioned with 10% polyacrylic acid, rinsed, and gently dried using cotton pellets. Restorations were placed under cotton roll isolation using hand-mixed high glass hybrid cement (HVGIC) (GC Gold Label Hybrid, GC Tokyo, Japan). A protective coating of petroleum jelly was applied to prevent early moisture contamination.

#### 2.6.3 Silver-modified atraumatic restorative treatment (SMART)

Cavities were cleaned using fluoridated toothpaste and a soft toothbrush. A 38% silver diamine fluoride (SDF) solution (Tedequim, Córdoba, Argentina) was applied to the carious dentin using a microbrush for 10–30 s. No mechanical removal of carious dentin or cavity preparation was performed.

Restorations were placed using the same HVGIC protocol as in the ART group, including cavity conditioning and moisture control. A protective petroleum jelly coating was applied after restoration placement.

### 2.7 Treatment delivery and clinical adaptations

A flow chart of the study is displayed in Figure 1. Depending on participant cooperation and clinical conditions, three scenarios were recorded:

1. Treatment completed under optimal clinical conditions;
2. Treatment modified to a less invasive approach due to clinical or behavioral limitations;
3. Inability to perform treatment, resulting in referral for care under sedation or general anesthesia.

### 2.8 Outcome measures

#### 2.8.1 Acceptability

Acceptability was defined as the participant's willingness to undergo the selected treatment. A YES/NO check-box was used to address whether the treatment was accepted or should the patient be referred to other dental behavior support strategy like conscious or deep sedation, or even general anesthesia.

#### 2.8.2 Feasibility

Feasibility was assessed by recording:

- Whether treatment was completed under optimal conditions;
- Occurrence of intra-operative difficulties;
- Completion of treatment according to protocol;
- Need to modify the treatment approach;
- Use of local anesthesia.

#### 2.8.3 Survival of restorations

Restoration survival was evaluated at 6 and 12 months by two independent, calibrated examiners (kappa ≥ 0.80). Evaluations were conducted using ART criteria and modified USPHS-Ryge criteria (19).

### 2.9 Statistical analysis

Data were summarized using means and standard deviations or frequencies, as appropriate. Chi-square tests were applied for categorical variables, with adjusted Monte Carlo simulations performed when necessary. Odds ratios, along with their corresponding confidence intervals, were estimated.

Restoration survival was analyzed using Kaplan–Meier survival curves. Given that more than one tooth could belong to the same patient, observations were not independent. To account for this clustering effect and to compare survival curves, Cox proportional hazards regression was performed using the patient as the clustering variable (20).

The level of statistical significance was set at α = 0.05. All analyses were conducted using SPSS version 20.0.

## 3 Results

### 3.1 Participant characteristics

A total of 150 patients attended the Special Care Dentistry Service during the study period, of whom 74 met the inclusion criteria and were enrolled (37 females, 37 males; mean age: 20.6 ± 11.2 years). The most frequent primary diagnoses were autism spectrum disorder (26%), intellectual disability (20%), epidermolysis bullosa (15%), Down syndrome (12%), and cerebral palsy (9%).

Patient characteristics according to medical condition, International Classification of Functioning, Disability and Health (ICF) classification, and complexity of care have been described in detail elsewhere (10). The ICF framework has direct implications for the domains included in the iADH Universal Case Mix Tool, which outlines the adaptations required for the provision of oral care to patients with disabilities. These domains encompass communication, behavioral support, medical status, oral risk factors, autonomy, legal considerations, and access to adapted care.

### 3.2 Care complexity and treatment allocation

The mean BDA Case Mix score was 16.8 ± 9.5, corresponding to moderate complexity. Distribution by category was: mild (*n* = 26), moderate (*n* = 24), severe (*n* = 22), and extreme (*n* = 2).

Significant differences in care complexity were observed among treatment groups. Participants treated with SMART presented significantly higher BDA scores compared to those treated with ART or CRT (OR = 3.730; 95% CI: 1.82–6.75; *p* ≤ 0.001) (Table 2).

**Table 2:** Distribution of care complexity (BDA).

Complexity level (BDA scores)	*n*	SMART	ART	CRT	GA
Standard (0)	0	0	0	0	0
Mild (1–9)	26	5	12	10	0
Moderate (10–19)	24	14	9	1	0
Severe (20–29)	22	14	5	0	0
Extreme (30+)	2	2	0	0	2
Total	74	35	26	11	2

Ref. *n* = number of patients.

Regarding iADH uCMT domains:

- “Communication” and “Behavioral Support” required fewer adaptations in the CRT group (*p* = 0.001)
- “Access to Care” required the greatest adaptations in ART and SMART groups

### 3.3 Acceptability of treatments

All selected treatments were accepted by participants. SMART was the most frequently chosen approach (*n* = 35), primarily due to fear of rotary instrumentation.

ART was selected by 26 participants, mainly due to intolerance to noise and vibration, limited cooperation, and the desire to avoid general anesthesia. CRT was chosen by 11 participants, typically those with lower complexity and prior positive dental experiences.

Two participants required referral for treatment under sedation or general anesthesia due to lack of acceptance of all in-clinic approaches.

### 3.4 Feasibility of treatment

Optimal clinical conditions were achieved in 75.47% of SMART restorations (40/53), 88.22% of ART restorations (30/34) and 100% of CRT restorations (13/13).

The main barriers to optimal treatment delivery were limited cooperation (50.5%), involuntary movements/spasticity (20.5%) and difficulty in moisture control (20%).

Local anesthesia was required in 8 ART cases, all CRT cases and none of the SMART cases.

### 3.5 Survival of restorations

#### 3.5.1 Six-month evaluation

At 6 months, 97 out of 100 restorations were classified as successful according to ART criteria. Three failures occurred in the SMART group due to marginal defects >0.5 mm requiring repair.

According to modified USPHS-Ryge criteria, minor changes in marginal discoloration, integrity, and surface texture were observed but were classified as clinically acceptable.

#### 3.5.2 Twelve-month evaluation

**Figure 2:**
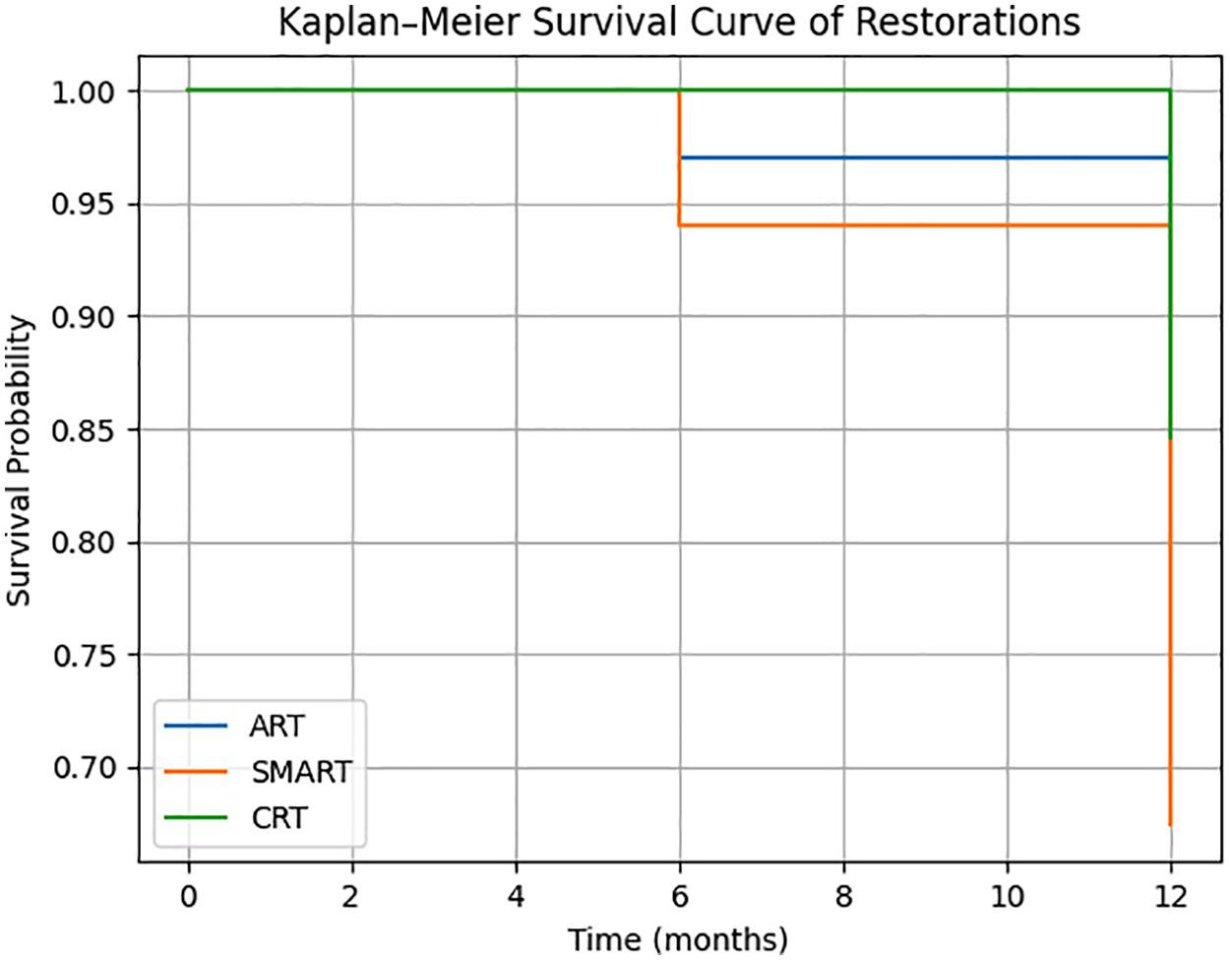
Kaplan–Meier survival curve.

At 12 months, 26 restorations failed according to ART criteria and 23 restorations failed according to USPHS-Ryge criteria (Figure 2).

Failure types, according to ART criteria (Table 3), were marginal defects >0.5 mm (15), wear >0.5 mm (1), secondary caries (2), complete loss of restoration (4), tooth exfoliation (1) and pulpal symptoms (3).

**Table 3:** Number of failed restorations in each cohort according to ART criteria.

Reason for failure	SMART	ART	CRT	Total
marginal defects >0.5 mm	12	3	0	15
wear >0.5 mm	1	0	0	1
secondary caries	0	0	2	2
complete loss of restoration	4	0	0	4
tooth exfoliation	1	0	0	1
pulpal symptoms	1	1	1	3
Total	19	4	3	26

According to USPHS-Ryge criteria, there were 18 failures in the SMART group, 3 failures in the ART group and 2 failures in the CRT group respectively.

#### 3.5.3 Survival rates

Out of the total number of treated teeth (*n* = 100), 10 were primary teeth and 90 were permanent teeth. No statistically significant differences were observed when correlating the survival of treatments in each cohort with the categories of primary vs. permanent teeth (*p* = 0.2).

Regarding the number of treated surfaces, cavities involving one surface (corresponding to Black's Class I and V) and two or more surfaces (Black's Class II and III) were identified; 9 restorations involved two surfaces, while 91 involved a single surface. The results also showed no statistically significant differences between treatment survival and the number of surfaces treated, possibly due to the low representation of multi-surface cavities in the evaluated sample (*p* = 0.1).

At 12 months, survival percentages were 90.3% for ART, 67.4% for SMART, and 84.3% for CRT according to USPHS-Ryge and 88.2% for ART, 64.1% for SMART, and 76.9% for CRT with significant differences between ART/CRT vs. SMART (*p* ≤ 0.01) according to ART criteria (Table 4). SMART showed significantly lower survival compared to ART and CRT (*p* < 0.001).

**Table 4:** Restoration survival percentages and number of failures at 12 months.

Treatment	Survival % Ryge criteria	Failures Ryge criteria	Survival % ART criteria	Failures ART criteria	*p* ≥ 0.05
ART	90.3	3	88.2	4	A
SMART	67.4	18	64.1	19	B
CRT	84.6	2	76.9	3	A
Total		23		26	

Ref. Different letters show statistically significant differences (Cox regression).

## 4 Discussion

This cohort study provides clinically relevant evidence on the performance of minimally invasive restorative approaches in patients with disabilities within a real-world Special Care Dentistry setting. Importantly, the findings extend beyond comparative effectiveness by highlighting how treatment selection, feasibility, and outcomes are intrinsically linked to patient complexity and contextual factors, supporting the need for structured, adaptive care frameworks.

A key strength of this study lies in its patient-centered design, in which participants and caregivers based treatment allocation on informed decision-making (21). While this resulted in heterogeneous cohorts, it reflects real clinical practice and underscores the importance of shared decision-making models in Special Care Dentistry. Within emerging frameworks of care, such models are increasingly recognized as essential components of ethical and effective treatment planning, particularly for individuals with cognitive or behavioral limitations (22, 23).

SMART was the most frequently selected approach, particularly among participants with higher BDA complexity scores. This highlights its relevance as an access-enabling intervention, especially in patients with severe behavioral limitations, reduced cooperation, or restricted access to advanced care modalities such as sedation or general anesthesia.

The use of the iADH Case Mix Tool provided additional insight into the determinants of treatment selection. Interestingly, the choice of SMART was more strongly associated with higher scores in domains such as “access to adapted care” rather than “behavior support”, “oral risk factors”, or “autonomy”. This suggests that patients opting for SMART were not necessarily those with the most challenging clinical or behavioral profiles, but rather those who are underserved or face barriers to accessing conventional dental care.

This finding underscores the importance of contextual and systemic factors in shaping treatment decisions and reinforces the need for adaptable care pathways in Special Care Dentistry. They also emphasize the need to move beyond a “one-size-fits-all” approach toward complexity-based clinical pathways, where treatment selection is guided not only by lesion characteristics but also by patient-specific factors, including functional status, behavioral profile, and access to care. In this context, tools such as the BDA Case Mix Tool and iADH uCMT demonstrated practical utility in stratifying patients and identifying domains requiring adaptation, supporting their integration into clinical decision-making frameworks.

In terms of effectiveness of the restorative options, the present results demonstrate that ART achieved high survival rates comparable to CRT, reinforcing its role as a reliable minimally invasive strategy across varying levels of care complexity. These findings are consistent with previous studies in populations with disabilities and medically compromised individuals, where ART has shown favorable long-term outcomes (24, 25). From a clinical excellence perspective, ART appears to offer an optimal balance between biological preservation, procedural simplicity, and durability.

These results also show that, while the SMART approach was feasible and could be implemented in patients with greater complexity, its survival rates were lower compared to conventional approaches. However, interpreting these outcomes solely as a reflection of treatment failure would be reductive. In high caries-risk patients, restorative survival is intrinsically influenced by underlying disease activity and patient-related factors (e.g., oral hygiene, parafunctional habits), and it is unrealistic to expect any restorative strategy to perform optimally in the absence of effective disease control measures.

SMART may still play a valuable role in arresting caries lesions, even in cases where restorations are classified as failures according to USPHS-Ryge or ART criteria. In this sense, its contribution should be understood within a broader disease management framework rather than being judged exclusively on restorative longevity. Within a broader innovation framework, SMART can be understood as a transitional or bridging strategy, facilitating initial disease control in otherwise hard-to-treat populations (26).

Despite its high acceptability and feasibility, SMART demonstrated significantly lower survival rates compared to ART and CRT. This finding raises important considerations regarding its role in long-term care planning. While previous studies have reported comparable or even superior outcomes for SMART in pediatric populations, the present results suggest that its effectiveness may be more limited in heterogeneous populations with disabilities, particularly those with higher functional complexity.

Most of the reasons for failure of SMART restorations were related to significant chipping or marginal defects >0.5 mm (12/19–63.16%). Following the one-year evaluation period, and in light of the lower survival rates observed in SMART restorations compared to ART restorations—despite both approaches utilizing high-viscosity GIC (HVGIC)—a mechanistic explanation was considered. In SMART procedures, carious tissue is not removed prior to restoration placement, which may result in a thinner and less mechanically supported GIC layer. This could increase susceptibility to marginal chipping or partial material loss over time, thereby affecting restoration survival.

Additionally, current evidence suggests that silver diamine fluoride (SDF) does not significantly enhance nor impair the adhesion of GIC to caries-affected dentin (27, 28). In contrast, the ART protocol includes selective removal of demineralized tissue and the application of 12% polyacrylic acid as a conditioner, which is known to improve the chemical bonding of GIC to the tooth substrate. These procedural differences may partially explain the disparities in clinical performance observed between the two approaches.

Clinical trials reporting comparative results among SMART, ART and CRT in vulnerable populations are scarce. In children and adult populations, a meta-analysis of four studies, comprising a total sample of 1,085 teeth, found no significant difference in the clinical effectiveness of SMART compared with ART for caries arrest at 12 months. Similarly, pooled data from two studies (*n* = 879 teeth) with a 24-month follow-up showed no significant difference between SMART and ART in children. In both analyses, the quality of evidence was rated as low (29).

The results of the present study differ from those reported for restorations of first primary molars treated using ART and SMART, where survival rates of 70% and 76.67% at 6 months, and 53.33% and 60% at 12 months, respectively, have been described. A key distinction between that clinical trial and our cohort study lies in the SMART implementation protocol (two sessions vs. a single session prior to restoration). In contrast, ART demonstrated superior performance compared with SMART in the present study. Nevertheless, failure rates for the SMART group were comparable between both studies at similar follow-up intervals (30).

From an innovation standpoint, the study highlights a critical tension between accessibility and durability. Minimally invasive techniques such as SMART improve access to care but may compromise long-term outcomes in certain subgroups. This trade-off underscores the importance of developing hybrid and staged treatment models, where initial minimally invasive interventions are followed by definitive restorative solutions when patient conditions allow (31).

Furthermore, the findings point toward opportunities for technological innovation, including the integration of digital workflows (e.g., CAD-CAM indirect restorations), advanced biomaterials with enhanced longevity, and telehealth-supported follow-up strategies. These approaches may help address current limitations in restoration survival while maintaining accessibility for patients with complex needs (31–33).

From an educational perspective, the results reinforce the need to train dental professionals in adaptive clinical reasoning, dental behavioral support, and the use of minimally invasive techniques within structured care frameworks. The ability to tailor treatment strategies according to patient complexity should be considered a core competency in modern Special Care Dentistry (34).

This study has some limitations. The non-randomized design and patient-driven treatment allocation introduce potential selection bias, although they enhance external validity. There is a potential for confounding by indication arising from the patient-driven allocation model. This methodological choice was intentional, as it reflects real-world clinical decision-making grounded in ethical principles of shared decision-making, particularly in contexts where access to sedation or general anesthesia is limited. In such scenarios, clinicians and caregivers must often prioritize treatment approaches that are feasible under existing constraints, even if these may not represent the most durable option under ideal conditions.

The observed association between treatment selection and patient complexity, as reflected by higher BDA scores in the SMART group, highlights a central and unavoidable challenge in Special Care Dentistry: the need to balance feasibility with effectiveness in highly heterogeneous and often vulnerable populations.

Survival percentages in the present study showed slight variations depending on the assessment criteria applied. These discrepancies were primarily attributed to pulpal involvement, which is considered only under ART criteria for restoration evaluation. Specifically, three restorations were classified as successful according to USPHS/Ryge criteria—based on marginal integrity, color stability, and anatomical form—yet exhibited symptoms requiring endodontic treatment. Consequently, these cases were deemed failures under ART criteria.

The relatively small sample size and unequal distribution across treatment groups may limit statistical power. Additionally, the follow-up period, while sufficient to detect early failures, does not allow for long-term survival assessment.

Future research should focus on longitudinal studies with larger samples, as well as the development and validation of integrated care models that combine clinical, behavioral, and technological components. Evaluating patient-reported outcomes, such as satisfaction and quality of life, will also be essential to fully assess the impact of these approaches.

## 5 Conclusions

SMART, while highly acceptable and accessible, showed significantly lower survival rates compared to ART and CRT after 12 months. ART remains a robust minimally invasive option across varying complexity levels.

The results support the need for personalized, complexity-based treatment planning and underscore the importance of balancing accessibility with long-term effectiveness in Special Care Dentistry.

## Data Availability

The raw data supporting the conclusions of this article will be made available by the authors, without undue reservation.
